# Strain-specific *Plasmodium falciparum* growth inhibition among Malian children immunized with a blood-stage malaria vaccine

**DOI:** 10.1371/journal.pone.0173294

**Published:** 2017-03-10

**Authors:** Matthew B. Laurens, Bourema Kouriba, Elke Bergmann-Leitner, Evelina Angov, Drissa Coulibaly, Issa Diarra, Modibo Daou, Amadou Niangaly, William C. Blackwelder, Yukun Wu, Joe Cohen, W. Ripley Ballou, Johan Vekemans, David E. Lanar, Sheetij Dutta, Carter Diggs, Lorraine Soisson, D. Gray Heppner, Ogobara K. Doumbo, Christopher V. Plowe, Mahamadou A. Thera

**Affiliations:** 1 Division of Malaria Research, Institute for Global Health, University of Maryland School of Medicine, Baltimore, Maryland, United States of America; 2 Malaria Research and Training Center, Bamako, Mali; 3 U.S. Military Malaria Vaccine Program, Walter Reed Army Institute of Research, Silver Spring, Maryland, United States of America; 4 GSK Vaccines, Rixensart, Belgium; 5 Malaria Vaccine Development Program, U.S. Agency for International Development, Washington, DC, United States of America; Ehime Daigaku, JAPAN

## Abstract

The blood-stage malaria vaccine FMP2.1/AS02A, comprised of recombinant *Plasmodium falciparum* apical membrane antigen 1 (AMA1) and the adjuvant system AS02A, had strain-specific efficacy against clinical malaria caused by *P*. *falciparum* with the vaccine strain 3D7 AMA1 sequence. To evaluate a potential correlate of protection, we measured the ability of participant sera to inhibit growth of 3D7 and FVO strains *in vitro* using high-throughput growth inhibition assay (GIA) testing. Sera from 400 children randomized to receive either malaria vaccine or a control rabies vaccine were assessed at baseline and over two annual malaria transmission seasons after immunization. Baseline GIA against vaccine strain 3D7 and FVO strain was similar in both groups, but more children in the malaria vaccine group than in the control group had 3D7 and FVO GIA activity ≥15% 30 days after the last vaccination (day 90) (49% vs. 16%, p<0.0001; and 71.8% vs. 60.4%, p = 0.02). From baseline to day 90, 3D7 GIA in the vaccine group was 7.4 times the mean increase in the control group (p<0.0001). In AMA1 vaccinees, 3D7 GIA activity subsequently returned to baseline one year after vaccination (day 364) and did not correlate with efficacy in the extended efficacy time period to day 730. In Cox proportional hazards regression models with time-varying covariates, there was a slight suggestion of an association between 3D7 GIA activity and increased risk of clinical malaria between day 90 and day 240. We conclude that vaccination with this AMA1-based malaria vaccine increased inhibition of parasite growth, but this increase was not associated with allele-specific efficacy in the first malaria season. These results provide a framework for testing functional immune correlates of protection against clinical malaria in field trials, and will help to guide similar analyses for next-generation malaria vaccines.

**Clinical trials registry**: This clinical trial was registered on clinicaltrials.gov, registry number NCT00460525.

## Introduction

The blood stage malaria vaccine FMP2.1/AS02A, comprised of recombinant 3D7 strain *Plasmodium falciparum* apical membrane antigen 1 (AMA1) and the adjuvant system AS02A, was tested in a Phase 2 clinical trial in 400 malaria-exposed Malian children aged 1–6 years. Vaccination according to a 0, 1, 2-month schedule did not show efficacy against the primary endpoint, but showed approximately 20% efficacy against first and multiple clinical malaria episodes defined using different parasite density thresholds, and 64.3% efficacy (p = 0.03) against clinical malaria caused by parasites with AMA1 corresponding to the 3D7 vaccine strain at pre-defined polymorphic amino acid sites [1]. As this was the first clinical trial of a blood stage malaria vaccine to show efficacy against clinical malaria in an endemic area, we assessed potential *in vitro* correlates of protection. In this Phase 2 trial, three doses of the AMA1 (FMP2.1/AS02A) vaccine induced high levels of anti-AMA1 antibody comparable to those found in semi-immune adults living at the site. The difference in anti-AMA1 antibody titers from baseline to the time point just before the first malaria episode was associated with a lower risk of clinical malaria (hazard ratio (HR) 0.72, p<0.001) [1].

Inhibition of merozoite invasion of erythrocytes as measured by a growth inhibition assay (GIA) represents a potential functional correlate of protection that has been studied in clinical trials of blood-stage malaria vaccines. GIA evaluates the functional activity of antibodies directed against blood stage antigens by measuring parasite growth in the presence of immune serum compared to non-immune serum. While previous studies have shown that antibody titers against blood stage *P*. *falciparum* malaria antigens correlate with serum inhibition of parasite growth [2–5] and that GIA corresponds to decreased risk of clinical malaria in children residing in malaria-endemic areas [6], other published studies have not found a correlation of GIA with clinical malaria risk [7–9], and a recent study found increased risk of clinical malaria with increasing GIA [10]. These seemingly discordant results could arise from association of GIA with both protection and exposure risk. Until now, the relationship of growth inhibition to protection against clinical malaria and the ability to kill the parasite *in vivo* have not been assessed for malaria vaccines based on blood-stage antigens like AMA1, because no previous blood-stage vaccine has demonstrated even partial protection against clinical malaria in field studies.

## Materials and methods

### Ethics statement

The trial was conducted in compliance with the International Conference on Harmonization of Good Clinical Practices, the Declaration of Helsinki and regulatory requirements of Mali. The study protocol and informed consent process were approved by the institutional review boards of the University of Sciences, Techniques and Technology Faculty of Medicine, Pharmacy and Dentistry in Bamako, Mali; the University of Maryland Baltimore; the Walter Reed Army Institute of Research; and the United States Army Surgeon General. Written informed consent was obtained prior to screening and enrollment. Verbal consent of illiterate parents or guardians was administered and then documented using their thumbprints, a process verified by signatures of independent witnesses.

### Clinical trial

Details of the Phase 2 trial are published elsewhere [1]. Briefly, 400 children in Bandiagara, Mali, where malaria has intense seasonal transmission, were randomized on a 1:1 basis to receive three monthly immunizations (days 0, 30 and 60) with either the AMA1 malaria vaccine or a control rabies vaccine, and followed actively and passively for one season for the primary endpoint of clinical malaria episode defined as fever of >37.5°C and a microscopically identified *P*. *falciparum* infection with a parasite density of 2,500/microliter or greater. Clinical malaria episodes were classified as having AMA1 sequence matching or not matching that of the vaccine strain 3D7 based on complete concordance with eight polymorphic amino acid positions in the cluster 1 loop of AMA1 domain I that were identified before the analysis as important in the development of strain-specific immunity against AMA1 based on both *in vitro* [11] and molecular epidemiological evidence [12].

### GIA

Strain-specific GIA was assessed for sera collected at baseline and at eight subsequent time points over the two year follow-up period, including study days 0, 30, 60, 90, 150 and 240 corresponding to the first malaria season following immunization, and days 364, 547, and 730 corresponding to the beginning, middle and end of the second post-immunization malaria season. Serum inhibition of the growth of *P*. *falciparum*, strain 3D7 and a genetically distant FVO strain, was assessed using a miniaturized, high-throughput method by laboratory staff who were blinded to treatment group. Samples were processed and evaluated for growth inhibitory activities by quantification of parasite lactate dehydrogenase (pLDH) in a 384-microtiter plate format [10,13]. In brief, serum samples were dialyzed to remove any drugs that may have anti-parasite activities. Next, sera were heat inactivated for 20 minutes at 56°C, cooled on ice, and pre-absorbed for 1 hour using 50% hematocrit red blood cells (RBCs; 5 μL RBCs for every 100 μL of sera) at room temperature with intermittent mixing. After the pre-absorption, samples were centrifuged for 2 minutes at 14,000*g* to pellet RBCs. The required amount of serum needed was removed without disruption of the RBC pellet and transferred to and diluted to a 20% concentration (vol/vol final). A 4% hematocrit parasite suspension (0.3–0.4% parasitemia) was added to the plates, and the plates were incubated for one cycle length (*P*. *falciparum* strain, 3D7 = 40 hours, FVO = 44 hours) gassed (5% CO_2_, 5% O_2_, 90% N_2_) at 37°C. Plates were harvested, processed for pLDH detection (lysing of parasites, adding a parasite specific LDH substrate) and optical density in each well was read at 650 nm. Children demonstrating ≥ 15% activity were considered responders, a reproducible cut-off value calculated based on the mean inhibition of U.S. non-immune pre-immune sera + 2 standard deviations.

Special considerations were made to quality control for plate variations by positioning negative and positive plate controls at various locations on the plate. Positioning the controls at opposite sides of each plate allowed monitoring of changing conditions during the enzymatic reaction (development of the plate). Every assay plate contained a complete set of assay controls; one of these assay controls is a historical reference sample (pooled anti-MSP1p42 FVO specific rabbit serum, tested at 10% serum concentration). The growth inhibitory activity of this control against 3D7 and FVO parasites had to fall within a pre-determined range of inhibition and served as pass/fail criterion for each plate. The analysis of the samples was done using the pre-immune samples of each subject to determine the individual sample baseline activity.

### Statistical methods

For both 3D7-specific and FVO-specific GIA results, means at various times since baseline and the mean difference in GIA results from baseline were compared between AMA1 and control vaccine recipients using Student’s t-test. Percentages of participants who met a minimum pre-specified threshold of 15% at each study time point, as well as percentages of participants who converted from a negative GIA at baseline (<15%) to a positive GIA (≥15%) at 30 days after the last vaccination, and at one and two years after the first vaccination, were compared using a chi-square test with no continuity correction. For each analysis of means or percentages, p-values are presented for 12 comparisons, along with an indication of whether the difference is significant at significance level 1–0.95^1/12^, which is the level required for an overall type I error rate of 0.05 if the tests are statistically independent; since there are substantial positive correlations between GIA results at different times, these adjustments are likely to be conservative, making the overall type I error rate < 0.05 within each table.

The relationship between GIA results and risk of clinical malaria was examined by estimating the hazard ratio for the first or only episode of clinical malaria with onset between day 90 and day 240 after the first vaccination in Cox proportional hazards regression models for risk of clinical malaria. These models included both GIA at the study time point just before the first clinical episode or the difference in GIA from baseline to the study time point just before the first clinical episode included in the model as a time-varying covariate.

Statistical analyses were done with SAS 9.2 and 9.3 statistical software (Cary, North Carolina, USA) and NCSS software (Number Cruncher Statistical Systems, Kaysville, Utah).

### Data availability

GIA data used in the analysis are accessible at http://dx.doi.org/10.7910/DVN/ZUQFHQ.

## Results

### Mean GIA activity against vaccine strain 3D7 was higher in the AMA1 vaccine group

**Fig 1:**
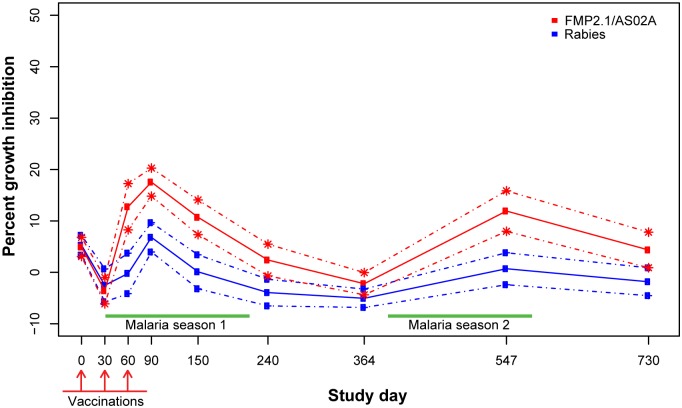
Mean 3D7 growth inhibition assay results by study day and group with 95% confidence intervals.

Before immunization, which began at the transition from the dry season to the malaria transmission season, mean 3D7 GIA activity was around 5% for sera from both AMA1 vaccinees and controls (Table 1). At study day 90, corresponding to 30 days after the last vaccination, mean 3D7 GIA activity had increased to 17.5% in the AMA1 group and 6.8% in the control vaccine group. From study day 60 through day 547, except for day 364, mean 3D7 GIA remained significantly higher in the AMA1 group when compared to the control group (Fig 1, Table 1) after adjustment for multiple comparisons. The increase in mean GIA activity measured 30 days after the last vaccination compared to baseline was 7.4-fold higher in the AMA1 group than in the control group (12.4% vs. 1.7%, p<0.0001). At the start of the second malaria season (study day 364), neither group had mean 3D7 GIA activity above zero. By the end of the second malaria season (study day 547), mean 3D7 GIA activity was again higher in the AMA1 group compared to the control group. At the start of the third malaria season (day 730), the AMA1 group displayed positive mean 3D7 GIA activity, but the control group did not (4.3% vs. -1.8%, p = 0.006). Mean GIA activity and differences from baseline in the two groups at time points from baseline to day 730 are summarized in Table 1.

**Table 1:** Mean 3D7 growth inhibition assay result, and change from baseline to 30 days after last vaccination and one and two years after enrollment, with standard deviation (SD), by vaccine group.

	Vaccine Group		
Study Day	AMA1 (SD)	Control (SD)	p-value^1^
0 (baseline)	4.9 (13.3)	5.3 (13.9)	0.81
30	-3.6 (18.0)	-2.5 (22.7)	0.61
60	12.8 (31.4)	-0.2 (27.6)	<0.0001^2^
90	17.5 (19.3)	6.8 (20.0)	<0.0001^2^
150	10.7 (23.8)	0.1 (23.0)	<0.0001^2^
240	2.4 (21.7)	-3.9 (18.0)	0.002^2^
364	-2.2 (15.2)	-5.1 (12.4)	0.047
547	11.9 (27.3)	0.7 (21.4)	<0.0001^2^
730	4.3 (23.6)	-1.8 (18.2)	0.006
90–0	12.4 (21.9)	1.7 (22.7)	<0.0001^2^
364–0	-7.3 (18.3)	-10.1 (18.4)	0.14
730–0	-0.3 (26.1)	-6.6 (20.9)	0.010

SD = standard deviation

^1^ Two-sided t-test

^2^ p ≤ α* = 1–0.95^1/12^ = 0.00427; assuming all 12 tests are independent, testing at significance level α* keeps the overall type I error rate at 0.05.

### GIA seropositivity and seroconversion against vaccine strain 3D7 were higher in the AMA1 vaccine group

Based on a predetermined threshold of 15% GIA, participants were classified as having significant GIA activity against the vaccine strain 3D7 or not. At baseline, both AMA1 vaccine and control groups were essentially equivalent with 15–16% in each group showing activity (Table 2). This percentage decreased at day 30 but increased by day 60 and peaked around day 90 (30 days after the last vaccination) in both groups. On subsequent days 150 and 240, the percentage of participants showing 3D7 GIA activity in each group decreased compared to the previous time point, but then increased during the second malaria season, which ended at approximately day 547. Compared to baseline, the proportion of participants showing positive GIA activity at the last study visit (day 730) was slightly larger for AMA1 vaccinees (17.7% vs. 14.4% for those with values at both times, exact p = 0.46 for two-sided McNemar test) but had decreased by about one-half for control vaccinees (7.3% vs. 14.7%, exact p = 0.035). Among those without GIA activity of at least 15% at study start, 46% “seroconverted” to a positive GIA response by day 90 in the AMA1 group while only 14% did so in the control group (Table 2; p<0.0001).

**Table 2:** Percentage of subjects with growth inhibition assay activity ≥15% against 3D7 parasites, and percentage seroconverting from below up to or above threshold 30 days after last vaccination and at one and two years after enrollment, by vaccine group.

	Vaccine Group		
Time point	AMA1 (%)	Control (%)	p-value^1^
Day 0	32/199 (16.1)	31/201 (15.4)	0.86
Day 30	15/194 (7.7)	16/195 (8.2)	0.86
Day 60	57/190 (30.0)	25/191 (13.1)	<0.0001^2^
Day 90	97/195 (49.7)	30/192 (15.6)	<0.0001^2^
Day 150	65/194 (33.5)	21/189 (11.1)	<0.0001^2^
Day 240	35/192 (18.2)	12/189 (6.3)	0.0004^2^
Day 364	24/191 (12.6)	9/187 (4.8)	0.008
Day 547	60/187 (32.1)	27/185 (14.6)	<0.0001^2^
Day 730	32/181 (17.7)	13/177 (7.3)	0.003^2^
Day 0 <15%GIA; Day 90 ≥15%GIA	75/163 (46.0)	23/163 (14.1)	**<**0.0001^2^
Day 0 <15%GIA; Day 364 ≥15%GIA	16/160 (10.0)	7/159 (4.4)	0.053
Day 0 <15%GIA; Day 730 ≥15%GIA	26/155 (16.8)	10/151 (6.6)	0.006

^1^ Two-sided t-test

^2^ p ≤ α* = 1–0.95^1/12^ = 0.00427; assuming all 12 tests are independent, testing at significance level α* keeps the overall type I error rate at 0.05.

### GIA activity against vaccine strain 3D7 was not associated with protection

For Cox regression modeling of clinical malaria with onset from day 90 to day 240, with either 3D7 GIA or the change in 3D7 GIA since the day of vaccination treated as a time-varying covariate, there were 61 first or only malaria episodes among 163 AMA1 vaccinees and 59 such episodes among 149 recipients of rabies vaccine. There was a suggestion in AMA1 vaccinees of an association between 3D7 GIA and an increased risk of clinical malaria (HR 1.014 for a change of 1% in 3D7 GIA, p = 0.023). A similar but slightly weaker association was seen with change in 3D7 GIA (HR 1.007, p = 0.23). There was no evidence of an association between 3D7 GIA and clinical malaria risk in recipients of rabies vaccine (HR 1.004, p = 0.59) or with change in 3D7 GIA (HR 0.999, p = 0.89).

During the first malaria season, only three first or only episodes of malaria identical to the vaccine strain occurred between days 90 and 240 among AMA1 vaccinees, and 13 such episodes occurred among recipients of rabies vaccine. We did not fit Cox models to malaria identical to the vaccine strain.

### GIA against non-vaccine strain FVO was similar in AMA1 and control vaccinees

**Fig 2:**
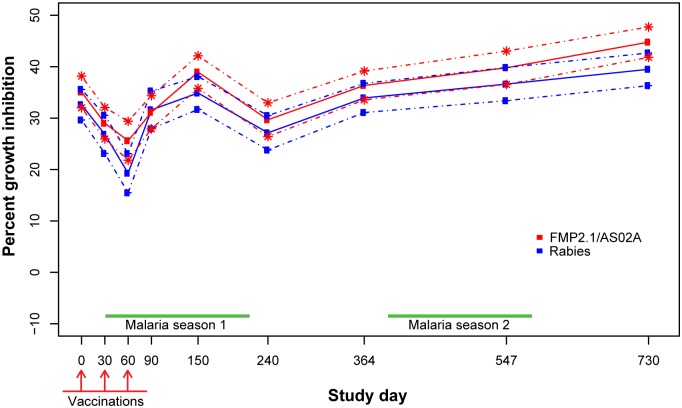
Mean FVO growth inhibition assay results by study day and group with 95% confidence intervals.

The mean baseline activity against FVO parasites was higher than the activity measured against 3D7 parasites. This activity reflects parasite inhibition induced by natural exposure and may target other blood stage antigens that are more cross-reactive than AMA1. At study start FVO GIA activity in AMA1 vaccinees averaged 35% and subsequently dropped. The activity recovered to 39% by day 150, corresponding to the peak of the first malaria season of follow-up. By day 240, FVO GIA values in AMA1 vaccinees had fallen to 30%, with subsequent measurements increasing up to the highest mean FVO GIA of 45% on day 730, the last day of follow-up (Fig 2, Table 3).

**Table 3:** Mean FVO growth inhibition assay result, and change from baseline to 30 days after last vaccination and one and two years after enrollment, with standard deviation (SD), by vaccine group.

	Vaccine Group		
Study Day	AMA1 (SD)	Control (SD)	p-value^1^
0 (baseline)	35.1 (22.0)	32.6 (21.4)	0.26
30	29.0 (21.6)	26.8 (26.3)	0.38
60	25.6 (26.5)	19.3 (26.7)	0.021
90	31.1 (22.9)	31.6 (25.8)	0.85
150	39.0 (22.3)	34.9 (22.3)	0.075
240	29.7 (23.0)	27.1 (23.3)	0.28
364	36.4 (19.7)	33.9 (19.7)	0.23
547	39.8 (22.5)	36.6 (22.3)	0.17
730	44.8 (20.1)	39.5 (21.3)	0.017
90–0	-3.8 (19.2)	-0.7 (20.6)	0.13
364–0	1.2 (26.6)	2.1 (25.3)	0.73
730–0	10.1 (27.7)	7.4 (25.8)	0.34

SD = standard deviation

^1^Two-sided t-test

^2^ p ≤ α* = 1–0.95^1/12^ = 0.00427; assuming all 12 tests are independent, testing at significance level α* keeps the overall type I error rate at 0.05. There were no such p-values in this table.

In the control group, mean GIA activity followed a similar pattern. Mean FVO growth inhibition was 33% at study start, with a subsequent fall to 19% on day 60, then an increase up to 35% on day 150 at the peak of the first malaria season. Average FVO GIA activity then dropped in control vaccinees to 27% at day 240, and subsequently increased to a peak value of 39% on the final study day, 730 (Fig 2, Table 3).

When comparing FVO GIA activity between the two groups, AMA1 vaccinee sera collected on day 60 showed slightly higher FVO GIA activity compared to the control group (25.6% vs. 19.3%, p = 0.021). By the next measurement at day 90, however, mean FVO GIA values were comparable in the two groups and were similar to baseline values (Table 3). The difference in FVO GIA activity from baseline (day 0) to the start of the second and third malaria seasons (days 364 and 730, respectively) showed that compared to baseline, both study groups had slightly higher FVO GIA activity at day 364 and somewhat higher activity at day 730. At the end of the study on day 730, AMA1 vaccinees had somewhat higher mean FVO GIA activity than the control group (44.8% vs. 39.5%, p = 0.017), but the difference was not significant after multiple comparison adjustment. The increases from baseline (10.1% and 7.4%, respectively) were not significant.

Table 4 shows the number and percent of participants in each group who achieved a positive GIA response against FVO parasites based on the predetermined threshold of at least 15%. At baseline, 79–80% of the individuals in each group displayed growth inhibitory activity. This percentage decreased at day 30 but subsequently increased on days 60, 90 and 150 in both groups. On day 240, the percentage in each group with anti-FVO GIA activity was lower compared to the previous measurement, but then the percentages increased to values of 93.9% in AMA1 recipients and 90.3% in rabies vaccine recipients on day 730. Interestingly, the percent positive for FVO GIA activity was somewhat greater in the AMA1 group than in the control group during the first malaria season (days 30, 60, 90, and 150) while values at the subsequent time points tended to be similar in the two groups, though always slightly higher for AMA1 vaccine recipients. Among those with FVO GIA activity <15% at study start, 45% in the AMA1 group and 33% in the control group had achieved the 15% GIA threshold by study day 90. These percentages increased to 83% and 71%, respectively, at study day 364 and to 89% and 85%, respectively, by study day 730. The proportions of participants in the two groups who had seroconverted did not differ significantly at day 90, day 364, or day 730.

**Table 4:** Percentage of subjects with growth inhibition assay activity ≥15% against FVO parasites, and percentage seroconverting from below up to or above threshold 30 days after last vaccination and at one and two years after enrollment, by vaccine group.

	Vaccine Group		
Time point	AMA1 (%)	Control (%)	p-value^1^
Day 0	159/199 (79.9)	158/201 (78.6)	0.75
Day 30	144/194 (74.2)	125/195 (64.1)	0.031
Day 60	123/190 (64.7)	100/191 (52.4)	0.014
Day 90	140/195 (71.8)	116/192 (60.4)	0.018
Day 150	170/194 (87.6)	151/189 (79.9)	0.040
Day 240	144/192 (75.0)	130/189 (68.8)	0.18
Day 364	168/191 (88.0)	161/187 (86.1)	0.59
Day 547	158/187 (84.5)	155/185 (83.8)	0.85
Day 730	170/181 (93.9)	159/176 (90.3)	0.21
Day 0 <15%GIA; Day 90 ≥15%GIA	18/40 (45.0)	14/42 (33.3)	0.28
Day 0 <15%GIA; Day 364 ≥15%GIA	33/40 (82.5)	29/41 (70.7)	0.21
Day 0 <15%GIA; Day 730 ≥15%GIA	34/38 (89.5)	33/39 (84.6)	0.53

^1^ Chi-square test with no continuity correction

^2^ p ≤ α* = 1–0.95^1/12^ = 0.00427; assuming all 12 tests are independent, testing at significance level α* keeps the overall type I error rate at 0.05. There were no such p-values in this table.

Results of Cox regression analyses of risk of clinical malaria with onset between day 90 and day 240 and FVO GIA in AMA1 recipients were similar to those for 3D7 GIA, but there was no suggestion of a significant association between risk of clinical malaria and FVO GIA or change from baseline in FVO GIA in either AMA1 or rabies vaccine recipients.

## Discussion

Overall, these results demonstrate highly significant GIA activity against vaccine strain 3D7 parasites in the serum of children vaccinated with the FMP2.1/AS02A vaccine as compared to control rabies vaccine. In contrast, there were only modest and usually non-significant differences in GIA activity of the two serum sets against FVO parasites. These GIA activities did not correlate with the observed significant efficacy of the vaccine against 3D7-like parasites [14], although the number of 3D7-like malaria events was too small to assess a possible association between 3D7 GIA activity in individual participants and protection against malaria due to 3D7-like parasites.

The current analysis reports results of serum growth inhibition, and differs from other published studies of growth inhibition using purified immunoglobulin G from plasma. The methodology used may influence results [15]. A study of clinically immune adults from western Kenya reported growth inhibition in 22 serum samples and enhancement of parasite growth in all IgG preparations from the same serum samples [16], while other studies have reported correlation using different methods to measure growth inhibition [17,18]. Although measurement of growth inhibition using purified IgG is currently viewed as a reference standard as followed by the PATH-Malaria Vaccine Initiative funded GIA reference center at the NIH, plasma samples were not collected in the current study for this purpose. Further, the potential for IgG purification methods to bias the immunoglobulin population [15] influenced the choice of dialyzed serum used for this analysis. A separate clinical trial of the same vaccine, both dose and adjuvant system, tested in malaria-naïve adults showed correlation of GIA using different methodologies at both the Walter Reed Army Institute of Research, where the current samples were tested, and at the NIH GIA reference center [19].

Baseline 3D7 GIA activity in this cohort of 400 children aged 1–6 years was similar to previously reported activities in semi-immune adults living at the same study site [20]. Thirty days after the last vaccination, GIA increased significantly only in AMA1 vaccinees. The initial increases in GIA waned during the second year of follow-up, although the mean decrease in GIA from baseline was lower in AMA1 vaccinees than in controls. This decline over time is consistent with other epidemiologic studies that found reduced GIA activity with increasing age in children [10,21,22], which may be explained by modification of functional antibody activity, changes in the fine specificity and/or avidity of the antibodies, or waning immune memory that is not subsequently boosted by natural exposure. Similar to our findings here, GIA has been associated with increased risk of malaria [10], and has not been associated with protection.

The decreased 3D7 GIA activity at the start of the second year of follow up is also consistent with the lack of any measurable overall or allele-specific efficacy against clinical malaria in the AMA1 vaccinees during this time period. Subsequent to the start of the second year of follow-up (study day 364), mean 3D7 GIA activity increased in both groups by the end of the second malaria season (study day 547), likely due to natural boosting of antimalarial immunity with natural exposure. In contrast to waning GIA, anti-AMA1 antibody levels peaked in the AMA1 group 30 days after vaccination and remained at a high level for the entire 2-year follow up period, while levels in the control group remained low [23]. These observations suggest that anti-AMA1 antibody levels measured by ELISA may be markers of exposure and vaccination.

Using a threshold of 15% 3D7 GIA to define a specific response to blood stage parasites, more AMA1 vaccinees than controls had positive GIA at all post-vaccination study time points after day 30, and AMA1 vaccinees were more likely to “seroconvert” from negative to positive GIA. Post-immunization absolute GIA and increases in GIA were consistent with allele-specific efficacy against clinical malaria in the first year. Although both GIA and anti-AMA1 antibody levels remained higher in the AMA1 vaccine group for two years after immunization, no allele-specific vaccine efficacy was demonstrated in the second year of follow-up [23].

Enhanced 3D7 GIA activity did not correlate with overall malaria risk due to both vaccine and non-vaccine strains in this study. It may be that GIA represents a surrogate measure of environmental exposure to and ongoing risk of malaria, similar to previous studies showing that anti-malarial immunity is associated with subsequent malaria risk because individuals with greater malaria exposure over time develop higher antibody levels [24].

To date, other clinical efficacy trials of blood stage malaria vaccines have not shown efficacy against clinical malaria in children, precluding investigations of potential correlates of protection. While we were able to determine that children in the AMA1 group had a higher GIA measurement post-vaccination compared to controls, no statistically significant evidence of allele-specific protection was demonstrated. This functional assay did not correlate with the protection demonstrated in the vaccine trial, but it remains possible that GIA may correlate with protection against other candidate blood stage malaria vaccines.

Correlation of 3D7 GIA activity with risks of clinical malaria in malaria vaccinees up to study day 240 showed a HR of 1.004 (p = 0.44), suggesting that the protection demonstrated in this group could be explained by other unknown factors. As it pertains to humoral immune responses, growth inhibition assays can only assess the biological function of antibodies. It is conceivable that AMA1 specific antibodies could display efficacy if acting in concert with phagocytic cells expressing Fc-receptors as has been shown for other blood stage antigens (e.g. MSP-3/GLURP) [25–29]. Alternatively, a role for AMA1 induced cell mediated immunity is consistent with its liver stage expression [30–31]. In conclusion, it is likely that an effective immune response against malaria will require both cell and antibody-mediated components.

The waning of allele-specific protection in the second year of follow-up occurred at the same time that 3D7 GIA activity in AMA1 vaccinees decreased compared to the initial follow-up period. This suggests that 3D7 GIA may be a surrogate of protection rather than a functional correlate. Perhaps a booster dose in AMA1 vaccinees just prior to the second malaria season would have increased immunity above a critical threshold necessary to provide the allele-specific protection observed during the first malaria season after vaccination.

Baseline mean FVO GIA activity in the study population was greater than the 20% previously measured in adults at the study site [20]. This observation is consistent with other reports of increased growth inhibitory activity in children compared to adults from the same geographic area [8,21], and suggests that the mechanism mediating protective antimalarial immunity may differ in adults compared to children. The lack of a boosting effect after vaccinations on study day 90 contrasts with adults receiving the same dose of the AMA1 vaccine in Bandiagara who showed significantly higher inhibition of FVO parasites compared to the control group after the last vaccine dose [20]. The increases in mean FVO GIA from baseline to the end of the study may reflect natural exposure as increases were seen in both study groups.

The observation that more AMA1 vaccinees had FVO GIA than did control vaccinees beginning with the time point after the first vaccination until 90 days after the last vaccination, suggests that immunization with one allele may broaden immune responses to other alleles.

## Conclusions

Vaccination with the AMA1 candidate malaria vaccine protein based on the 3D7 *P*. *falciparum* amino acid sequence increased strain-specific inhibition of parasite growth as measured by GIA against the 3D7 strain of *P*. *falciparum*. This increase was not associated with a reduced risk of clinical malaria caused by parasites with vaccine strain-type AMA1 in the two malaria seasons following vaccination. Additional immunologic analyses including affinity-purification of anti-AMA1 antibodies with subsequent characterization of these immunoglobulins regarding fine specificity, antibody isotype and avidity may increase our understanding of the functional basis of this allele-specific vaccine-induced immunity. Moreover, the antibody, GIA and efficacy data indicate that annual booster immunizations may be required to maintain protective immunity. The current study suggests that GIA is not an immune correlate of strain-specific protection against clinical malaria for this AMA1 vaccine, but assessment of GIA to other candidates may still provide information that can help guide the development of more broadly protective next-generation malaria vaccines.

## References

[1] Thera MA, Doumbo OK, Coulibaly D, Laurens MB, Ouattara A, Kone AK, et al (2011) A field trial to assess a blood-stage malaria vaccine. N Engl J Med 365: 1004–1013. 10.1056/NEJMoa1008115 21916638 PMC3242358

[2] Aoki S, Li J, Itagaki S, Okech BA, Egwang TG, Matsuoka H, et al (2002) Serine repeat antigen (SERA5) is predominantly expressed among the SERA multigene family of Plasmodium falciparum, and the acquired antibody titers correlate with serum inhibition of the parasite growth. J Biol Chem 277: 47533–47540. 10.1074/jbc.M207145200 12244052

[3] Malkin EM, Diemert DJ, McArthur JH, Perreault JR, Miles AP, Giersing BK, et al (2005) Phase 1 clinical trial of apical membrane antigen 1: an asexual blood-stage vaccine for Plasmodium falciparum malaria. Infect Immun 73: 3677–3685. 10.1128/IAI.73.6.3677-3685.2005 15908397 PMC1111886

[4] Mullen GE, Ellis RD, Miura K, Malkin E, Nolan C, Hay M, et al (2008) Phase 1 trial of AMA1-C1/Alhydrogel plus CPG 7909: an asexual blood-stage vaccine for Plasmodium falciparum malaria. PLoS ONE 3: e2940 10.1371/journal.pone.0002940 18698359 PMC2491586

[5] Miura K, Zhou H, Diouf A, Moretz SE, Fay MP, Miller LH, et al (2009) Anti-apical-membrane-antigen-1 antibody is more effective than anti-42-kilodalton-merozoite-surface-protein-1 antibody in inhibiting plasmodium falciparum growth, as determined by the in vitro growth inhibition assay. Clin Vaccine Immunol 16: 963–968. 10.1128/CVI.00042-09 19439523 PMC2708396

[6] Crompton PD, Miura K, Traore B, Kayentao K, Ongoiba A, Weiss G, et al (2010) In vitro growth-inhibitory activity and malaria risk in a cohort study in Mali. Infect Immun 78: 737–745. 10.1128/IAI.00960-09 19917712 PMC2812204

[7] Marsh K, Otoo L, Hayes RJ, Carson DC, Greenwood BM (1989) Antibodies to blood stage antigens of Plasmodium falciparum in rural Gambians and their relation to protection against infection. Trans R Soc Trop Med Hyg 83: 293–303. 2694458 10.1016/0035-9203(89)90478-1

[8] McCallum FJ, Persson KE, Mugyenyi CK, Fowkes FJ, Simpson JA, Richards JS, et al (2008) Acquisition of growth-inhibitory antibodies against blood-stage Plasmodium falciparum. PLoS ONE 3: e3571 10.1371/journal.pone.0003571 18958278 PMC2570221

[9] Perraut R, Marrama L, Diouf B, Sokhna C, Tall A, Nabeth P, et al (2005) Antibodies to the conserved C-terminal domain of the Plasmodium falciparum merozoite surface protein 1 and to the merozoite extract and their relationship with in vitro inhibitory antibodies and protection against clinical malaria in a Senegalese village. J Infect Dis 191: 264–271. 10.1086/426398 15609237

[10] Bejon P, Cook J, Bergmann-Leitner E, Olotu A, Lusingu J, Mwacharo J, et al (2011) Effect of the pre-erythrocytic candidate malaria vaccine RTS,S/AS01E on blood stage immunity in young children. J Infect Dis 204: 9–18. 10.1093/infdis/jir222 21628653 PMC3105039

[11] Dutta S, Lee SY, Batchelor AH, Lanar DE (2007) Structural basis of antigenic escape of a malaria vaccine candidate. Proc Natl Acad Sci U S A 104: 12488–12493. 10.1073/pnas.0701464104 17636123 PMC1941496

[12] Takala SL, Coulibaly D, Thera MA, Batchelor AH, Cummings MP, Escalante AA, et al (2009) Extreme polymorphism in a vaccine antigen and risk of clinical malaria: Implications for vaccine development. Sci Transl Med 1: 2ra5. 10.1126/scitranslmed.3000257 PMC2822345 20165550

[13] Duncan EH, Bergmann-Leitner ES. Miniaturized growth inhibition assay to assess the anti-blood stage activity of antibodies. Methods Mol Biol. 2015;1325:153–65. 10.1007/978-1-4939-2815-6_13 26450387

[14] Ouattara A, Takala-Harrison S, Thera MA, Coulibaly D, Niangaly A, Saye R, et al (2013) Molecular basis of allele-specific efficacy of a blood-stage malaria vaccine: vaccine development implications. J Infect Dis 207: 511–519. 10.1093/infdis/jis709 23204168 PMC3537449

[15] Bergmann-Leitner ES, Mease RM, Duncan EH, Khan F, Waitumbi J, Angov E. Evaluation of immunoglobulin purification methods and their impact on quality and yield of antigen-specific antibodies. Malar J 7:129 10.1186/1475-2875-7-129 18625058 PMC2490700

[16] Shi YP, Udhayakumar V, Oloo AJ, Nahlen BL, Lal AA (1999) Differential effect and interaction of monocytes, hyperimmune sera, and immunoglobulin G on the growth of asexual stage Plasmodium falciparum parasites. Am J Trop Med Hyg 60: 135–141. 9988337 10.4269/ajtmh.1999.60.135

[17] Bergmann-Leitner ES, Duncan EH, Mullen GE, Burge JR, Khan F, Long CA, et al (2006) Critical evaluation of different methods for measuring the functional activity of antibodies against malaria blood stage antigens. Am J Trop Med Hyg 75: 437–442. 75/3/437 [pii]. 16968918

[18] Perraut R, Marrama L, Diouf B, Sokhna C, Tall A, Nabeth P, et al (2005) Antibodies to the conserved C-terminal domain of the Plasmodium falciparum merozoite surface protein 1 and to the merozoite extract and their relationship with in vitro inhibitory antibodies and protection against clinical malaria in a Senegalese village. J Infect Dis 191: 264–271. 10.1086/426398 15609237

[19] Spring MD, Cummings JF, Ockenhouse CF, Dutta S, Reidler R, Angov E, et al (2009) Phase 1/2a study of the malaria vaccine candidate apical membrane antigen-1 (AMA-1) administered in adjuvant system AS01B or AS02A. PLoS ONE 4: e5254 10.1371/journal.pone.0005254 19390585 PMC2669163

[20] Thera MA, Doumbo OK, Coulibaly D, Diallo DA, Kone AK, Guindo AB, et al (2008) Safety and immunogenicity of an AMA-1 malaria vaccine in Malian adults: results of a phase 1 randomized controlled trial. PLoS ONE 3: e1465 10.1371/journal.pone.0001465 18213374 PMC2186380

[21] Dent AE, Bergmann-Leitner ES, Wilson DW, Tisch DJ, Kimmel R, Vulule J, et al (2008) Antibody-mediated growth inhibition of Plasmodium falciparum: relationship to age and protection from parasitemia in Kenyan children and adults. PLoS ONE 3: e3557 10.1371/journal.pone.0003557 18958285 PMC2570335

[22] McCallum FJ, Persson KE, Mugyenyi CK, Fowkes FJ, Simpson JA, Richards JS, et al (2008) Acquisition of growth-inhibitory antibodies against blood-stage Plasmodium falciparum. PLoS ONE 3: e3571 10.1371/journal.pone.0003571 18958278 PMC2570221

[23] Thera MA, Coulibaly D, Kone AK, Guindo AB, Diallo DA, Traore K, et al (2010) Extended safety, immunogenicity and efficacy of WRAIR's AMA1 malaria vaccine (FMP2.1) adjuvanted in GSK biologicals' AS02A in 1–6 year old children in Bandiagara, Mali. Am J Trop Med Hyg 85: 354–401.

[24] Greenhouse B, Ho B, Hubbard A, Njama-Meya D, Narum DL, Lanar DE, et al (2011) Antibodies to Plasmodium falciparum antigens predict a higher risk of malaria but protection from symptoms once parasitemic. J Infect Dis 204: 19–26. 10.1093/infdis/jir223 21628654 PMC3105040

[25] Oeuvray C, Bouharoun-Tayoun H, Gras-Masse H, Bottius E, Kaidoh T, Aikawa M, et al (1994) Merozoite surface protein-3: a malaria protein inducing antibodies that promote Plasmodium falciparum killing by cooperation with blood monocytes. Blood 84: 1594–1602. 8068948

[26] Bouharoun-Tayoun H, Oeuvray C, Lunel F, Druilhe P (1995) Mechanisms underlying the monocyte-mediated antibody-dependent killing of Plasmodium falciparum asexual blood stages. J Exp Med 182: 409–418. 7629503 10.1084/jem.182.2.409 PMC2192140

[27] Bouharoun-Tayoun H, Attanath P, Sabchareon A, Chongsuphajaisiddhi T, Druilhe P (1990) Antibodies that protect humans against Plasmodium falciparum blood stages do not on their own inhibit parasite growth and invasion in vitro, but act in cooperation with monocytes. J Exp Med 172: 1633–1641. 2258697 10.1084/jem.172.6.1633 PMC2188756

[28] Oeuvray C, Theisen M, Rogier C, Trape JF, Jepsen S, Druilhe P (2000) Cytophilic immunoglobulin responses to Plasmodium falciparum glutamate-rich protein are correlated with protection against clinical malaria in Dielmo, Senegal. Infect Immun 68: 2617–2620. 10768952 10.1128/iai.68.5.2617-2620.2000 PMC97467

[29] Theisen M, Soe S, Oeuvray C, Thomas AW, Vuust J, Danielsen S, et al (1998) The glutamate-rich protein (GLURP) of Plasmodium falciparum is a target for antibody-dependent monocyte-mediated inhibition of parasite growth in vitro. Infect Immun 66: 11–17. 9423833 10.1128/iai.66.1.11-17.1998 PMC107852

[30] Chuang I, Sedegah M, Cicatelli S, Spring M, Polhemus M, Tamminga C, et al (2013) DNA prime/Adenovirus boost malaria vaccine encoding P. falciparum CSP and AMA1 induces sterile protection associated with cell-mediated immunity. PLoS ONE 8: e55571 10.1371/journal.pone.0055571 23457473 PMC3573028

[31] Schwenk R, Banania G, Epstein J, Kim Y, Peters B, Belmonte M, et al (2013) Ex vivo tetramer staining and cell surface phenotyping for early activation markers CD38 and HLA-DR to enumerate and characterize malaria antigen-specific CD8+ T-cells induced in human volunteers immunized with a Plasmodium falciparum adenovirus-vectored malaria vaccine expressing AMA1. Malar J 12: 376 10.1186/1475-2875-12-376 24168370 PMC3819688

